# Inequalities in the Ability for People With Type 2 Diabetes and Prediabetes to Adapt to the Reduction in In-Person Health Support and Increased Use of Digital Support During the COVID-19 Pandemic and Beyond: Qualitative Study

**DOI:** 10.2196/55201

**Published:** 2024-06-25

**Authors:** Sophie Turnbull, Christie Cabral

**Affiliations:** 1 Bristol Medical School Population Health Sciences University of Bristol Bristol United Kingdom; 2 Centre for Academic Primary Care Bristol Medical School Population Health sciences, University of Bristol Bristol United Kingdom

**Keywords:** diabetes, diabetic, DM, diabetes mellitus, type 2 diabetes, type 1 diabetes, prediabetes, prediabetic, COVID-19 pandemic, COVID-19, SARS-CoV-2, coronavirus, severe acute respiratory syndrome, coronavirus infections, novel coronavirus, motivation, health inequalities, self-care, mHealth, mobile health, app, apps, application, applications, digital health, digital intervention, digital interventions, telemedicine, telehealth, virtual care, virtual health, virtual medicine, remote consultation, telephone consultation, video consultation, remote consultations, telephone consultations, video consultations

## Abstract

**Background:**

The COVID-19 pandemic created unprecedented challenges for people with type 2 diabetes (T2D) and prediabetes to access in-person health care support. Primary care teams accelerated plans to implement digital health technologies (DHTs), such as remote consultations and digital self-management. There is limited evidence about whether there were inequalities in how people with T2D and prediabetes adjusted to these changes.

**Objective:**

This study aimed to explore how people with T2D and prediabetes adapted to the reduction in in-person health support and the increased provision of support through DHTs during the COVID-19 pandemic and beyond.

**Methods:**

A purposive sample of people with T2D and prediabetes was recruited by text message from primary care practices that served low-income areas. Semistructured interviews were conducted by phone or video call, and data were analyzed thematically using a hybrid inductive and deductive approach.

**Results:**

A diverse sample of 30 participants was interviewed. There was a feeling that primary care had become harder to access. Participants responded to the challenge of accessing support by rationing or delaying seeking support or by proactively requesting appointments. Barriers to accessing health care support were associated with issues with using the total triage system, a passive interaction style with health care services, or being diagnosed with prediabetes at the beginning of the pandemic. Some participants were able to adapt to the increased delivery of support through DHTs. Others had lower capacity to use DHTs, which was caused by lower digital skills, fewer financial resources, and a lack of support to use the tools.

**Conclusions:**

Inequalities in motivation, opportunity, and capacity to engage in health services and DHTs lead to unequal possibilities for people with T2D and prediabetes to self-care and receive care during the COVID-19 pandemic. These issues can be addressed by proactive arrangement of regular checkups by primary care services and improving capacity for people with lower digital skills to engage with DHTs.

## Introduction

Type 2 diabetes (T2D) is a chronic disease that affects a large number of people and creates a significant burden for patients and the health services that support them. In the United Kingdom, 1 in 10 people older than 40 years now has T2D and around a third of adults living in England have prediabetes [1]. Prediabetes puts individuals at high risk of developing T2D and the associated health complications [2], including cardiovascular pathologies, kidney disease, eye problems, and foot ulcers [3].

The COVID-19 pandemic created unprecedented challenges for people with T2D and prediabetes to access in-person health care and self-care support in the United Kingdom [4,5]. Routine checkup appointments and nonurgent hospital care were cancelled due to government-implemented social distancing rules and the reallocation of health services and personnel to manage COVID-19 patients [6]. There was a 77% reduction in the number of tests for hemoglobin A_1C_ in the United Kingdom between March and December 2020, which provides an objective marker of glycemic levels and diabetes disease status [7]. Primary care teams were required to accelerate plans to increase the implementation of digital health technologies (DHTs), such as remote consultations and digital self-management [5]. Concurrently, face-to-face community-based interventions (eg, Healthier You service) transitioned to fully remote digital delivery [8].

There is conflicting evidence about the impact of the reduction of in-person health care and increased digital support on health inequalities during the COVID-19 pandemic. In this paper, we use the term “health inequality” in the sense used by Marmot [9,10] in his key papers on this topic to denote differences in health due to social determinants such as neighborhood deprivation. There is qualitative evidence that people in the United Kingdom with T2D faced varied challenges in health care access; some struggled to contact health care professionals (HCPs), while others noticed no change [11]. The difference in experience accessing care may relate to the individuals’ ability to adjust to the increased delivery of health care through digital and remote approaches. In a qualitative study with HCPs working in primary and secondary care during the COVID-19 pandemic, the HCPs felt that while most of their patients were able to adapt to the change in the delivery of services (because they had no alternative options), they had concerns about digital exclusion for those who were older, less physically fit, or from lower socioeconomic groups [12]. A YouGov survey from 2020 indicated that older individuals (older than 55 years), those with a carer, or those who were unemployed were more likely to have negative experiences with DHTs than the general population [13]. A qualitative study found no barriers to DHT use among people with T2D during the COVID-19 pandemic [11]. Conversely, they reported that several had felt that their skills and confidence to use digital platforms to communicate with HCPs increased during pandemic, due to the increased prevalence of these digital tools in all areas of life (eg, work, social, and health) [11]. However, the study had limitations, as the sample were younger (79% were younger than 65 years) than the overall UK population (47% were younger than 65 years), and no information was provided about socioeconomic status (SES) [11].

As we move beyond the COVID-19 pandemic, we will also move into a new chapter in the delivery of in-person health care and self-care support and the use of DHTs [14]. The pandemic accelerated innovation in health care, and a Department of Health and Social Care white paper proposed that these advances should be made permanent [15]. Primary care clinicians have cited concerns that the lack of face-to-face appointments during the lockdown phases of pandemic resulted in poorer control of blood glucose and resulted in many people with prediabetes crossing the threshold into a T2D diagnosis [14]. Health services recovery plans have sought to address the backlog in care by retaining some digitally led tools that were used during the pandemic, including blended digitally enabled triage (remote tools and digital methods) [16], blended consultations (remote and face-to-face) [17,18], digital self-care tools such as remote-monitoring devices, and web-based support tools including the Healthy Living platform [14]. However, it is widely reported that there are continued challenges for patients accessing health care services, particularly in primary care [16]. It is essential that we understand the barriers that patients face when accessing and using these DHTs. This will support the identification of those who may need support to benefit from increasingly digitally led health care services.

We conducted a qualitative interview study to explore how people with T2D and prediabetes adapted to the reduction in in-person health and self-care support and the increased provision of support through DHTs during the lockdown stages of the COVID-19 pandemic and beyond. The interviews were conducted in April 2022, a total of 8 months after the final lockdown concluded in the United Kingdom (July 2022) and after emergency measures had been relaxed. This allowed us to capture reflections on experiences of the emergency stage of the COVID-19 pandemic and the transition into the recovery stage for health services, with the associated shifts in provision of services through DHTs. We wanted to explore issues with inequalities in access to support and any barriers or supporting factors to individuals with T2D and prediabetes adapting to the changes in access to support.

## Methods

A qualitative interview design was used [19]. We have adhered to the COREQ (Consolidated Criteria for Reporting Qualitative Research) reporting checklist [20].

### Ethical Considerations

All activities were approved by and conducted in accordance with the Health and Social Care Research Ethics Committee B, who granted a favorable ethical opinion on January 11, 2022 (reference 21/NI/02022), and the Declaration of Helsinki. The participants received both written and verbal information about the research. Informed consent was collected from all participants. Interview participants provided written consent before the interview was arranged, which was confirmed with verbal consent immediately prior to the interview.

### Participant Recruitment

A purposive sample of patients with T2D and prediabetes was recruited, which was diverse with respect to SES, gender, ethnicity, and age. Two primary care practices were selected to ensure access to a diverse patient population. Eligible patients were identified by staff in the recruited primary care practices by searching patient records for adults who were recorded as having a T2D or prediabetes diagnosis. A text message was sent out to eligible patients through the practice messaging system inviting them to enter the study. More than 90 potential participants expressed an interest in being interviewed. Interviewing continued until data saturation was reached and no new data arose in relation to the key themes, with a final sample of 30 participants.

### Data Collection Procedure

Participants were provided with written information about the study in advance and either completed the eConsent form or provided detailed verbal consent that was audio recorded before beginning the interview.

The interviews were semistructured and conducted by 1 researcher (ST). The topic guide (available in Multimedia Appendix 1) was developed by ST and CC, informed by the literature and the authors’ prior qualitative work on access to DHTs for people with T2D [21,22]. There were 3 iterations of the topic guide, with minor changes to questions about the potential of an intervention to reduce inequalities in access to DHTs, and around any unmet information needs they had about their condition. Field notes were taken during and after interviews. Participants were asked to describe their age range, gender, ethnicity, and occupation (or most recent employment if they were not currently employed). Their SES was determined by coding the occupational group using the Office of National Statistics Standard Occupational Classification 2020 [23] and mapping them to the 3 National Statistics Socio-economic Classification (NS-SEC) categories using the guidelines provided by the Office of National Statistics [24]. Interviews were recorded with consent on an encrypted audio-recorder and transferred to the University of Bristol secure servers. They were transcribed and uploaded to NVivo (Version 1.6.2; Lumivero) for analysis [25].

### Analysis

Thematic analysis was used [26], and data collection and analysis was iterative to allow emerging themes to be explored in subsequent interviews. ST initially took an inductive approach, allowing the themes to emerge from the data, and then took a deductive approach, organizing the themes into 2 broad preconceived concepts related to the study aims of exploring challenges in accessing health care and changes in the use of DHTs [19]. The codes were developed by 2 researchers working independently with the data to ensure a robust analysis. ST developed the initial coding structure, which CC then applied to a sample of transcripts independently. The final coding structure was agreed by consensus and applied to the whole data set (the coding tree is available in Multimedia Appendix 1). Participants were provided with a summary of the findings.

### Research Team and Reflexivity

#### Personal Characteristics

ST is a mixed-methods researcher with a BSc degree in psychology, MSc degree in neuropsychology, and a PhD degree in the impact of digital interventions on health inequalities for chronic conditions. CC is a senior researcher with a PhD degree in social anthropology and research projects in the fields of primary care, social care, and global health.

#### Relationship With Participants

There was no prior relationship with the study participants before the study commenced. All but 1 of the interviews were conducted over the phone, so participants would not have had any awareness of ST’s physical characteristics. They would have known that ST was a female researcher working at the University of Bristol. The participants knew that the study was about how people who are at risk or diagnosed with T2D use technology to help them with their health, fitness, or well-being. The position taken by the ST was that DHTs have the potential to improve access to health care support, but that it is likely that not all social groups will be able to benefit from these types of innovations in health care without support. This may have influenced the conduct of the interviews and interpretation of findings. However, care was taken to phrase questions openly and avoid leading participants, and we therefore believe these findings to be a credible representation of participants’ views.

## Results

### Sample

A total of 30 people were interviewed, who were diverse with respect to gender, age, type of T2D (diagnosed with T2D or prediabetes), ethnicity, and SES (Table 1). Although the majority (23/30, 77%) felt that they were able to navigate technology, the sample included those with no internet access and those with low digital skills (Table 1). Interviews lasted between 14 minutes and 1 hour 15 minutes.

**Table 1:** Sample sociodemographic information.

Sociodemographic information			Participants (n=30), n (%)
**Gender**			
	Female	15 (50)	
**Age range (years)**			
	20-29	1 (3)	
	30-39	1 (3)	
	40-49	8 (26)	
	50-59	7 (23)	
	60-69	8 (26)	
	70-79	2 (6)	
	80-89	3 (10)	
**Ethnicity**			
	African	1 (3)	
	Asian British	2 (6)	
	British African	1 (3)	
	Indian	3 (10)	
	White British	19 (63)	
	White European	2 (6)	
	White Irish	2 (6)	
**Type of diabetes**			
	T2D^a^	12 (40)	
	At risk of T2D	18 (60)	
**NS-SEC^b^ 3 classes based on current or previous occupation**			
	1. Managerial, administrative, and professional occupations	6 (20)	
	2. Intermediate occupations	7 (23)	
	3. Routine and manual occupations	12 (40)	
	Unemployed or long-term sick	4 (13)	
	Not possible to classify (religious sister)	1 (3)	
**SES^c^ group**			
	Low	14 (47)	
	Medium	9 (30)	
	High	6 (20)	
	Not possible to classify (religious sister)	1 (3)	
**Digital skills and access**			
	Generally confident using technology	23 (77)	
	Temporarily did not have access to the internet but had good digital skills	1 (3)	
	Not confident using technology but had devices they could use	5 (17)	
	Did not have internet connection or devices to access the internet and felt like they were unable to learn about new technology	1 (3)	
	Did not have internet connection or devices to access the internet and knew about bursaries they could use to access devices and the internet but felt that they were getting everything they needed without it	1 (3)	

^a^T2D: type 2 diabetes.

^b^NS-SEC: National Statistics Socio-economic Classification.

^c^SES: socioeconomic status.

### Results From Thematic Analysis

There were 2 broad groups of themes: challenges with accessing health care, and changes in the use of DHTs during and beyond the pandemic lockdown periods. An outline of the themes and subthemes is available in Table 2.

**Table 2:** Themes and subthemes.

Theme	Subtheme
Accessing health care services	Accessing primary care Perceptions of changes support for T2D^a^ and prediabetes Impact of patient engagement strategy on access to care Differences between people with prediabetes and T2D
Changes in the use of DHTs^b^	Impact of previous experience of DHTs on engagement Capability to use DHTs Opportunity to access DHTs

^a^T2D: type 2 diabetes.

^b^DHT: digital health technology.

### Accessing Health Care

#### Overview

Participants described a reduction in access to primary care services and increased provision of remote services. They had different perceptions of how support for their T2D or prediabetes had changed and used either passive or active engagement strategies in response to the changes in care, which impacted the level of support they received from primary care. Those with prediabetes appeared to experience a greater reduction in support, which led to increased engagement and interest in DHTs.

#### Access to Primary Care: “I Just Give Up. I Don’t Bother Anymore...”

Participants described difficulties in accessing primary care since the beginning of the pandemic. Some described how the phone triage systems setup during the pandemic had led to primary care feeling like “a complete closed-door system,” because trying to get an appointment “could take between 80 and 100 phone calls, whilst getting cut off” (ID A, male, T2D).

Those who reported having less free time or flexibility to call the practices in the morning and wait in a queue reported having challenges booking checkups, appointments, or blood tests using the total triage system:

...it’s just such a nightmare at the moment, trying to get an appointment...you have to ring at 8:00 in the morning...I’m just a bit hectic at the moment, I’ve got a new-born baby...

ID B, male, prediabetes

#### Perceptions of Changes in Health Care Support for T2D and Prediabetes

Participants had different perceptions of how support from HCPs for T2D and prediabetes changed during the pandemic. For some, diabetes support from HCPs continued as before and they “never had any problems” accessing care (ID R, female, T2D). Others spoke about how their health care support did continue, but checkups were “not as regular” (ID E2, male, prediabetes). Others described how health support from the National Health Service (NHS; eg, diabetes nurses and dieticians) completely stopped during the pandemic:

...[care] was really excellent up until the pandemic...everything got cut off as soon as lockdown started.

ID J2, male, prediabetes

#### Impact of Patient Engagement Strategy on Level of Health Care Support

Whether the participants had a passive or active engagement strategy with health services determined the level of care they received during the pandemic. The strategy was determined by their beliefs about how they should engage with the NHS during the pandemic and their entitlement to care. Those who took a passive approach (rationing or delaying seeking support) held the belief that they should not burden the NHS with non–COVID-19–related issues. They were “embarrassed to ring up the doctor because they’re so busy with important stuff...like COVID” (ID J, female, prediabetes). A man with T2D spoke about how he felt that access was limited, and he needed to ration requests for support for the care he needed most help with:

...didn’t feel my situation was important enough...the access was limited, so I had to be very picky about keeping up with my medication reviews, my physical review. I just felt like I needed to keep those going and not put any more pressure on the NHS.

ID A, male, T2D

Others took an active approach and requested appointments. They described contacting the practice due to the belief that if they were “not determined enough” (ID N2, male, prediabetes), they would not receive support for their condition. One woman described how her role in social care has meant she knew what help she was entitled to, which meant she proactively sought the care she felt she deserved:

...I am the one who pushes it, you see? I am the one who insists that I want support, because I know the system...because of social care [job] I know what is happening and what I can get or what I can't get.

ID D, female, T2D

#### Differences Between People With Prediabetes and T2D

Participants with both T2D and prediabetes experienced a reduction in health care support during the pandemic. However, those diagnosed with prediabetes around the beginning of the pandemic described how they had “no follow-up visit, appointments, information, nothing” (ID M, male, prediabetes). This led to confusion about how they should manage their condition and whether they still had the diagnosis.

Several of the participants with prediabetes spoke about how they wanted to have a blood glucose monitor at home, to keep track of their condition and so that they were no longer reliant on the health service to understand how their health condition was progressing:

I want to get one [blood glucose monitor] because I want to know what my level is, and then I can check in, in a couple of weeks to see if it’s actually going down or going up...instead of waiting however long to get an appointment with the doctor...

ID S, male, prediabetes

### Changes in the Use of DHTs

Participants described how positive or negative experiences of using DHTs and capability to use DHTs influenced their engagement with DHTs following the removal of in-person health care and self-care support.

#### Impact of Experience of DHTs on Engagement

Some participants had positive experiences of remote or web-based health care or exercise support, which supported further engagement. For example, a woman described how she “found it easier” requesting support through eConsultations, because she was able to write about her multiple and complex issues in her own time: “they only get five minutes with you face-to-face, but online, you can write down whatever you want” (ID F, female, prediabetes). Some participants reflected on how closures of gyms had prompted them to buy fitness watches “to monitor [their] fitness level” (ID D, female, T2D), or to seek out web-based fitness classes to keep them motivated to exercise. A woman with T2D spoke about how having access to some web-based support from her tai chi instructor led her to explore other support for her diabetes online: “I went on to look at something that he [tai chi instructor] had set for us to do, I then found other things and thought, ‘Oh, that looks interesting,’ and then I went on from there” (ID G2, female, T2D).

Negative experiences were linked to disengagement from DHTs. Participants stopped using DHTs because they hurt themselves, preferred in-person support, felt demotivated by the feedback from DHTs, or lost money by accidently subscribing to services they did not want. A woman with prediabetes explained that she had received remote support from a personal trainer, but “when you’re not face to face and we’re going on a video, you can’t do it...they gave me backache. So I’ve stopped” (ID C, female, prediabetes). She also reflected that remote support could not replace the in-person support in gyms “Because it’s in a group and it’s a lot of motivation...you push yourself...going to the gym and in itself is good because you know...it releases endorphins...” (ID C). Another woman with prediabetes described how there was no time to prepare for the shift to digital support from her exercise class, and she was not interested in replacing in-person with digital support: “I don’t use that kind of technology” (ID Q2, female, prediabetes). One man with prediabetes stopped tracking his exercise and movements during the pandemic using his fitness watch, because he was moving less and found the feedback highlighted “the lack of progress” (ID J2, male, prediabetes). A woman with T2D had a fall pendant and started receiving calls she had not asked for: “I was receiving calls [fall service] twice a week. They’d go, ‘Are you fine’ ‘Yes, I’m fine.’ And when I got my bank statement I found they took £60 out of my account for every phone call. They were ringing up, and I didn’t authorise it...” (ID K, female, T2D).

#### Capability to Use DHTs

Several participants described barriers to accessing and using DHTs caused by their capability (skills) to use these technologies. These included challenges finding web-based support that suited their individual needs, low digital skills, and the absence of good-quality support to use DHTs. Participants spoke about how they “get in a terrible mess” (ID Q2, female, prediabetes) when trying to use technology generally and did not know how to use emails, apps, or navigate the internet. One woman spoke about how the absence of good-quality instructions created a barrier for her taking her own blood pressure reading during the pandemic in her general practitioner practice:

[I] just kept reading the leaflet there, and then I just couldn’t—I just could not. I had a go at wrapping it, and the lady said, “No, you’ve got to do it yourself.”...I just walked out the building and I cried...That was the worst feeling I’ve had, like illiterate feeling, at 60.

ID J, female, prediabetes

The majority of participants who struggled with digital skills described how they were able to overcome issues by being shown how to use DHTs through videos or in-person support: “I’d got to ask my niece how to download the COVID-19 app for me because I couldn’t do it, I couldn’t understand it” (ID K, female, T2D). A young woman with prediabetes spoke about how she struggled to “access it [digital support] until I’ve been explained how to use it...if you can send me a video, show me how to do it before I do it, then it would be easier” (ID V, female, prediabetes). Another woman with T2D spoke about the importance of being shown how to use DHTs rather than having it done for them, so they could learn for themselves: “...[young people] don’t show you. They do it for you...But of course, where does that leave you? You’re going to ask all over again” (ID H, female, T2D). One man felt that he was not able to learn how to use technology generally or DHTs, even with support from others: “I haven’t got the brain to use it...The people are trying to teach me...I just give up” (ID F2, male, T2D).

The capacity to use DHTs was also impacted by a lack of awareness of what DHTs were available. For example, none of the participants who had been told that they were at risk of T2D spoke about being offered the web-based Healthier You program and were not aware of it when they were explicitly asked.

#### Opportunity to Access and Use DHTs

There were barriers related to the opportunity for participants to use DHTs caused by the cost of the internet access and DHTs. Two men spoke about how they were “not online” because they were retired and the internet was “just another bill...” they could not afford because they have other priorities, such as running a car (ID E2, male, prediabetes). A woman with prediabetes spoke about how she wanted a blood glucose monitor but “can’t afford that...” (ID F, female, prediabetes).

## Discussion

### Summary of Findings

There was evidence of inequalities in the ability for people with T2D and prediabetes to adapt to the reduction in access to in-person health care and self-care support and increased implementation of DHTs during the pandemic. There was an indication that those with prediabetes were more likely than those with T2D to feel that they had a lack of support from HCPs, particularly those who received their diagnosis near the beginning of the pandemic.

There was a near universal perception of a reduction in access to primary care services and a mixed perspective of the change in NHS support to manage T2D and prediabetes. Barriers to accessing primary care were greater for those who had less free time or flexibility to call the practices in the morning and wait in a queue for an appointment. The level of support provided for people with T2D or prediabetes was determined in part by the engagement strategy used by the patient. Those who contacted their health care provider about needing more support subsequently received it, while those who waited to be contacted received a lower level of support. Participants with both T2D and prediabetes experienced a reduction in health care support during the pandemic. However, those who were diagnosed with prediabetes around the beginning of the pandemic described how they had not received any follow-up care from health professionals. This led to confusion about how they should manage their condition and whether they still had the diagnosis. They spoke about wanting to have an at-home blood glucose monitor so that they would not be reliant on the NHS to track the progress of their condition. Prior positive or negative experiences of using DHTs impacted motivation to engage in this support during the COVID-19 pandemic. Those with less opportunity (eg, financial resources) and capability (digital skills, knowledge of what was available, and support to use DHTs) struggled to engage in health services delivered through technology.

### Strengths and Weaknesses

Several qualitative studies have explored the impact of COVID-19 lockdowns on people with T2D’s access to health care support [11,27-29]. However, none have explored the perspectives of people with T2D as we move beyond the emergency to the recovery stage of the pandemic, or the perspectives of people with prediabetes.

In this study, we explored inequalities in access to support to adapt to the changes brought about by the COVID-19 pandemic and therefore purposively recruited people with lower SES. People with lower SES have a higher incidence and severity of T2D [1,30,31]. They also faced greater barriers accessing in-person support [10-12] and DHTs prior to the pandemic [32-36]. Therefore, we selected a recruitment method that would increase the chance of including people from these groups. We asked 2 practices serving lower-income areas in Bristol to identify adults with a T2D or prediabetes diagnosis from their patient database and to send them a text message invitation. More than 90 people contacted the study team to express an interest in being involved. This study successfully recruited a sample that was diverse in respect to SES, gender, age, type of T2D (diagnosed or at risk), ethnicity, and digital skills. Just less than half (14/30, 47%) of the sample were from the low SES group, using the NS-SEC 3 classes of SES based on current or previous occupation [24]. Those with lower digital skills reported that the reason they engaged with the study was because the invitation text message included a phone number to contact the study team.

We acknowledge that this method of recruitment is biased toward people who have some interaction with primary care services. However, for this project, we wanted to recruit people who had been diagnosed with T2D or prediabetes to establish how support from primary care changed throughout the pandemic and how people responded to a shift in delivery of care through DHTs. Although we had planned resources for a translator, none of the participants requested this support. The study invitations were sent in English and did not include details about the availability of a translator due to limited space to include study details in the text message invitation. This may have acted as a barrier to participating in the study for people whose first language is not English.

Participants were offered interviews by video call and telephone. All but 1 participant selected telephone interviews. Complete audio data were recorded for all interviews, and there were no issues with lost data. In 2 of the telephone interviews, other people were present in the room where the interview was taking place, and 1 interview was conducted while the participant was driving. This could have impacted the interview content. The participants were not asked to comment on the transcripts. Multiple views of the data were conducted to promote confidence in the credibility of the findings [37]. To ensure that the coding scheme was robust, CC double coded a subset of interviews and ongoing discussion about coding structure was conducted. The authors ensured that a diverse range of experiences and opposing sides of arguments were identified and presented.

### Interpretations in the Context of Existing Literature

The findings from this study agree with previous evidence that during the emergency stage of the COVID-19 pandemic, people with T2D perceived primary care support to be less accessible (UK survey) [38] and had mixed experiences of access to health care support for diabetes (UK qualitative study) [11].

Our study adds to the mixed evidence of the acceptability and accessibility of increased delivery of health care through DHTs during the pandemic. A UK-based survey of patients with a range of conditions indicated that those from underserved populations (older, unemployed, with a carer) were more likely to report negative experiences of using DHTs during the pandemic [13]. A qualitative study found that some people with T2D had reported increased digital skills acquired through the pandemic due to the pervasive nature of digital platforms to communicate in all areas of people’s lives [11]. None of their participants in this study reported barriers to accessing DHTs. This may have been related to their sample being younger (79% were younger than 65 years) than the overall UK population (47% were younger than 65 years). A measure of SES was not provided, but it may be possible that the sample were from higher SES groups, which is associated with higher level of digital skills [39]. Our study agreed with the finding by Morris et al [40] that people with chronic conditions with greater access to resources (social, financial, digital skills, and knowledge) were better able to adapt their self-care routines to the reduction of support and the increased delivery of services through DHTs during the pandemic.

This replicates the authors’ prepandemic qualitative study of people with T2D, which found that technical proficiency and cost were barrier to the use of self-care DHTs, but that participants were able to draw from resources in their social networks to overcome these barriers [22]. This study also confirms findings of a qualitative study conducted prior to and during the pandemic with primary and secondary care clinicians, where they had concerns that some of their patients were excluded from support delivered by DHTs during the pandemic due to lower digital skills or lack of affordability of internet access [12].

Although we did not set out to apply the COM-B model in analysis, the 3 elements that are needed for behavior change proposed in the COM-B model have been identified in our study as important influences for adapting to the changes of the COVID-19 pandemic [41]. The COM-B model specifies that capability, opportunity, and motivation have to be present for a behavior to occur [41]. Those who had the opportunity and capability to engage with the total triage systems to access health care, or who were highly motivated to ensure that they received a higher level of health care support, were able to access greater support from health care services during the COVID-19 pandemic. Negative experiences of using DHTs reduced participants’ motivation to use web-based tools, and a lower capacity to use DHTs prevented participants from being able to use them.

### Implications for Practice and Policy

#### Improving Equality in Access to Health Care

The findings from this study have highlighted how procedures implemented during the pandemic created uneven access to health care. Participants described the “total triage” model system making primary care feel like a “closed door system” where some patients have given up seeking support. They described waiting in phone lines all day and not being able to access appointments, and a system where those who are able to phone early or who are most persistent are able to get appointments. Moving beyond the emergency stage of the COVID pandemic, the total remote triage is being replaced with a blended model where traditional methods are being used alongside digital tools [14,42]. The addition of digital triage may reduce barriers to accessing primary care services by providing those who are unable to wait in phone queues with an alternative method of seeking support. However, this will be beneficial only for those who are willing and able to use digital tools.

#### Improving Access to Monitoring of Disease Progression

There are concerns about the significant reduction in hemoglobin A_1C_ tests in the United Kingdom (77% between March and December 2020) during the pandemic and how this may result in suboptimal management of T2D [7]. Population-based studies have found that the completion of a higher number of primary care–based process checks for people with T2D results in lower rates of mortality, amputations, and emergency hospital admissions [43]. There were indications in this study of unequal access to care and checkups for people with T2D and prediabetes. This seemed to have a particular impact on those who had been diagnosed with prediabetes around the beginning of the pandemic, with no follow-up support from primary care. Some subsequently purchased their own blood glucose monitors, but others were not able to afford them. The COVID-19 pandemic has galvanized the push to supply more continuous blood glucose monitors to people with type 1 diabetes; however, this is not yet the case for people with T2D [14]. It is therefore essential that regular checkups are uniformly reestablished for people with both T2D and prediabetes as soon as possible to prevent the widening of health inequalities [43]. Those with prediabetes in this study did not report being aware of or offered access to the Healthy Lives program. Greater dissemination of the Healthier Lives program and other self-care support to people with prediabetes may reduce confusion around how to self-manage their condition [14].

#### Improving Access to DHTs

The Department of Health and Social Care plans to make the increased use of digital innovations since the beginning of the pandemic permanent [15]. The NHS Long Term Plan also laid out the ambition to provide a “digital first” health care service within the next 10 years [44]. Although many participants in this study responded positively to the increased use of DHTs to deliver health care, some reported barriers to accessing this support, caused by negative experiences of using DHTs, lower levels of digital skills, lack of access to the internet, and a preference for in-person support. It is essential that unliteral adoption of DHTs by patients is not assumed, and face-to-face services are still offered for those who are not able or willing to use DHTs. There is evidence that engagement with DHTs can be improved in lower SES groups using multimodal content and the provision of in-person support [45,46]. This was supported by our findings, where participants spoke about how support being shown how to use DHTs through videos, or in-person reduced barriers to use for those with lower digital skills. A scheme piloted by NHS digital found that the digital champions successfully supported people with lower digital skills to access to DHTs [47,48]. This model shows promise as a route to tackle inequalities in access to DHTs in the future.

### Conclusions

There was evidence of inequalities in the ability for people with T2D and prediabetes to adapt to changes in health care support and increased implementation DHTs during the pandemic. Those who reported having challenges accessing to health care support had greater barriers engaging with the total triage system, a passive interaction style, or a prediabetes diagnosis at the beginning of the pandemic. Adaptation to the increase in provision of support through DHT was determined by whether the participants had previous positive or negative experiences of DHTs, and whether they had the capacity (eg, digital skills, finances, and technical support) to access and use DHTs. Inequalities in motivation and opportunity to self-care can be addressed by increasing the visibility of self-care support and proactive arrangement of regular checkups by primary care services (thereby avoiding the use of triaging systems) for people with prediabetes and T2D. Digital champions show promise for improving capacity for people with lower digital skills to engage with DHTs.

## Appendix

Multimedia Appendix 1 Topic guides and coding tree.

Multimedia Appendix 2 COREQ (Consolidated Criteria for Reporting Qualitative Research) checklist.

## Abbreviations

COREQ: Consolidated Criteria for Reporting Qualitative Research
DHT: digital health technology
HCP: health care professional
NHS: National Health Service
NS-SEC: National Statistics Socio-economic Classification
SES: socioeconomic status
T2D: type 2 diabetes

## Data Availability

Anonymized data sets used and/or analyzed during this study are available from the corresponding author on reasonable request.
