# Supplementary material for: Inequalities in the Ability for People With Type 2 Diabetes and Prediabetes to Adapt to the Reduction in In-Person Health Support and Increased Use of Digital Support During the COVID-19 Pandemic and Beyond: Qualitative Study
Source: JMIR Diabetes. 2024 Jun 25;9:e55201. doi: 10.2196/55201 (PMC11234064; doi:10.2196/55201)
Supplement: Multimedia Appendix 1 [file diabetes_v9i1e55201_app1.docx]

This document contains the topic guides from the interviews and the coding tree from the analysis conducted in Nvivo.

## Topic guides

### Topic guide for interviews with people with T2D (v 1.0)

***Introduction***

- Thanks, introduce self and re-state the purpose of the interview.
- Check consent verbally – check information sheet has been read & if not go over key points then:
- You agree to our conversation being audio recorded?
- You know you are free to stop the interview at any point and you may skip questions you would prefer not to answer?
- Participant information: age, gender, employment, ethnic background

      ----------------------------------------------------------------------------------------------------------------------

1. Background: Could you start by telling me a bit about your experience of being diagnosed with T2D.
2. How are you currently managing your Diabetes?

- Probing themes of:
- Treatment (biomed & CAM) & self-care practice
- Do you feel well supported by the Nurse/GP/hospital?
- Where do you seek Diabetes care/support?
- Have you been referred to any services/support
- Been offered any courses – in person or online?

1. Can you tell me a bit about the technology you have used for health, fitness or wellbeing??
2. Prior to being told you had T2D or in response to it?
3. What is your reason for seeking out the health technology?
4. How often use
5. How did you first learn about the health technology you use?
6. Did you find any good resources to help you choose health technology?
7. Or did anyone help you?
8. Face any challenges accessing or using the technology you wanted to try?
9. Anything you have stopped using? Why?
10. Facilitators & barriers to engagement: what did you like/not like about using them?
11. How did COVID pandemic affect things for you?

- Probing themes of:
- change in support from health professionals
- change in use health technology

1. I am looking to develop a resource to support people to use health technology when they are first told they are at risk or diagnosed with T2D.
2. What would you have found helpful to support you to use health technology when you were first diagnosed?
3. Anything you think we should include
4. Brief overview of our intervention idea- ask for thoughts.
5. Anything else you would like to tell me?

### Topic guide for interviews for people at risk of T2D (v 1.0)

***Introduction***

- Thanks, introduce self and re-state the purpose of the interview.
- Check consent verbally – check information sheet has been read & if not go over key points then:
- You agree to our conversation being audio recorded?
- You know you are free to stop the interview at any point and you may skip questions you would prefer not to answer?
- Participant information: age, gender, employment, ethnic background

-----------------------------------------------------------------------------------------------------

1. Background- Could you start by telling me a bit about your experience of being told you were at risk of T2D?
2. Have you done anything differently since you were told you were at risk of developing T2D?

- Probing themes of ‘Healthy lifestyle’ changes, diet and exercise

1. What support have you been offered by your nurse /GP

- Feel well supported?
- Been offered any courses – in person or online?

1. Can you tell me a bit about the technology you have used for health, fitness or wellbeing?
2. Prior to being told you were at risk of T2D or in response to it?
3. What is your reason for seeking out the health technology?
4. How often use
5. How did you first learn about the health technology you use?
6. Did you find any good resources to help you choose health technology?
7. Or did anyone help you?
8. Face any challenges accessing or using the technology you wanted to try?
9. Anything you have stopped using? Why?
10. Facilitators & barriers to engagement: what did you like/not like about using them?
11. How did COVID pandemic affect things for you?

- Probing themes of:
- change in support from health professionals
- change in use health technology

1. I am looking to develop a resource to support people to use health technology when they are first told they are at risk or diagnosed with T2D.
2. What would you have found helpful to support you to use health technology when you were first told you were at risk of developing T2D?
3. Anything you think we should include
4. Brief overview of our intervention idea- ask for thoughts.
5. Anything else you would like to tell me?

## Coding tree

| Name | Description | Files | References |
| --- | --- | --- | --- |
| COVID |  | 0 | 0 |
| Access to health care |  | 0 | 0 |
| COVID did or didn't change access or support from HCPs |  | 22 | 32 |
| COVID risk in hospital setting |  | 2 | 2 |
| Didn't contact HCP because didn't feel their needs were important enough | During pandemic reluctant to contact HCP because they didn’t feel their needs were important enough. | 4 | 6 |
| Made health services harder to access |  | 12 | 20 |
| Not able to get diagnosis |  | 1 | 1 |
| Remote delivery made getting help less accessible | e.g. person with anxiety struggling on phone appointments and not able to take someone in with them to support them in appointments | 1 | 1 |
| Change in use of health tech |  | 1 | 1 |
| Can't use online appointments |  | 1 | 1 |
| Health tech used during covid caused health issues |  | 1 | 2 |
| Instructions too complicated |  | 1 | 2 |
| Started or stopped using tech because of covid |  | 12 | 15 |
| Provides covid pass |  | 1 | 1 |
| Impact on diabetes management |  | 0 | 0 |
| Adapting management strategies to COVID restrictions |  | 3 | 3 |
| Believe COVID had negative impact on diabetes |  | 1 | 1 |
| COVID created challenges for diabetes management activities |  | 11 | 15 |
| External motivators |  | 0 | 0 |
| Having a dog supported fitness in pandemic |  | 1 | 1 |
| Importance of maintaining routine in pandemic |  | 1 | 1 |
| Management activities better during covid |  | 6 | 7 |
| Stopped doing management activities because of fear of covid |  | 3 | 3 |
| Impact on life or wellbeing |  | 0 | 0 |
| Did not create major lifestyle changes |  | 2 | 2 |
| Negative impact |  | 1 | 1 |
| Concerns about protecting loved ones |  | 1 | 1 |
| COVID increased isolation |  | 8 | 13 |
| COVID infection impacted health |  | 1 | 1 |
| Had to shield |  | 2 | 3 |
| Nervous to go out |  | 2 | 2 |
| Stopped being able to work or changed role |  | 4 | 7 |
| More pressure at work |  | 1 | 1 |
| Stressful |  | 1 | 1 |
| Positive impact |  | 0 | 0 |
| Enjoyed the chance to slow down |  | 2 | 2 |
| Provided new job role |  | 1 | 1 |
| Re-evaluated priorities |  | 1 | 1 |
